# 7α-acetoxy-6β-hydroxyroyleanone (Roy) modulates IL-6/STAT3/JAK2 mRNA expression and suppresses tumor growth in glioblastoma cell models

**DOI:** 10.3389/fphar.2026.1728792

**Published:** 2026-02-13

**Authors:** Mariana Magalhães, Renato Spigarelli, Eva María Domínguez-Martín, Lino Ferreira, Thomas Efferth, Patrícia Rijo, Enzo Spisni, Célia Cabral

**Affiliations:** 1 PhD Programme in Experimental Biology and Biomedicine (PDBEB), Institute for Interdisciplinary Research, University of Coimbra, Coimbra, Portugal; 2 CNC-UC - Center for Neuroscience and Cell Biology, University of Coimbra, Coimbra, Portugal; 3 Coimbra Institute for Clinical and Biomedical Research (iCBR), Clinic Academic Center of Coimbra (CACC), Faculty of Medicine, University of Coimbra, Coimbra, Portugal; 4 CiBB - Centre for Innovative Biomedicine and Biotechnology, University of Coimbra, Coimbra, Portugal; 5 Department of Biological, Geological and Environmental Sciences, University of Bologna, Bologna, Italy; 6 Universidad de Alcala, Facultad de Farmacia, Departamento de Ciencias Biomédicas e Instituto de Investigación Química “Andrés M. del Río” (IQAR), Área de Farmacología, Grupos “Diseño, interacción y síntesis de compuestos biológicamente activos (DISCOBAC)” y “Bases moleculares de la resistencia en cáncer (CARE)”, Ctra. Madrid-Barcelona (Autovía A2), Alcalá de Henares, Madrid, Spain; 7 CBIOS Lusófona’s Research Center for Biosciences and HealthTechnologies, Campo Grand, Lisbon, Portugal; 8 Faculty of Medicine, University of Coimbra, Coimbra, Portugal; 9 Department of Pharmaceutical Biology, Institute of Pharmaceutical and Biomedical Sciences, Johannes Gutenberg University, Mainz, Germany; 10 Research Institute for Medicines (iMed.ULisboa), Faculty of Pharmacy, Universidade de Lisboa, Lisbon, Portugal; 11 Centro de Química Estrutural, Institute of Molecular Sciences, Universidade de Lisboa, Campo Grande, Coimbra, Portugal; 12 Faculty of Medicine, Instituto de Histologia e Embriologia, University of Coimbra, Rua Larga, Edifício da FMCU, Coimbra, Portugal; 13 Centre for Functional Ecology --Science for people and the planet (CFE), Department of Life Sciences, University of Coimbra, Coimbra, Portugal

**Keywords:** 3D cell models, 7α-acetoxy-6β-hydroxyroyleanone (Roy), antitumor activity, glioblastoma, tumor microenvironment

## Abstract

**Introduction:**

Glioblastoma (GB) is the most aggressive primary glioma, with a median survival of 15-18 months. Current treatments are often ineffective, largely due to tumor heterogeneity and recurrence. Advances in understanding GB’s molecular landscape and microenvironment have highlighted new therapeutic strategies to fight this life-threatening tumor. Given the pivotal role of natural compounds in drug discovery, those with anti-inflammatory and cytotoxic/cytostatic properties are emerging as promising candidates for GB therapy.

**Methods:**

This study investigates the antitumor and immunomodulatory effects of 7α-acetoxy-6β-hydroxyroyleanone (Roy), a diterpene isolated by our team from Plectranthus hadiensis Schweinf., using both 2D and 3D GB cell models. U87 cells were used as a standard GB model and to generate monocellular and multicellular spheroids (U87, HMC3, and/or HBMEC cells). Both models were treated with 16 µM of Roy, a concentration previously shown to be tumor-specific.

**Results:**

Roy significantly reduced spheroid size and metabolic activity over time, with the most pronounced effects observed in multicellular spheroids. This compound also inhibited cell proliferation by preventing colony formation and downregulating CDK4 and VEGFA mRNA levels. Roy’s bioactivity was enhanced in the presence of conditioned medium (secretome from GB and/or microglia cells), exerting a neuromodulatory effect by modulating IL6/JAK2/STAT3 mRNA expression and by suppressing the secretion of cytokines involved in the chronic inflammatory state within the GB microenvironment. Importantly, Roy was also able to cross the blood-brain barrier.

**Conclusion:**

These findings, in line with our previous work, underscore the cytotoxic potential of this natural compound, suggesting Roy as a promising lead candidate for future GB treatment strategies.

## 1 Introduction

Glioblastoma (GB), a grade 4 adult-type diffuse glioma, stands as the most deadly primary tumor of the central nervous system (CNS) (Louis et al., 2021). Latest statistics from the Global Cancer Observatory (GLOBOCAN) 2022 reveal that brain tumors account for more than 320,000 new cases and approximately 250,000 deaths per year worldwide (Ferlay et al., 2024). This devastating tumor typically results in a limited overall survival (OS), rarely exceeding 15–18 months, and a dismal 5-year survival rate (ca 6%), even with the established standard of care, which involves surgical resection, followed by chemoradiotherapy and adjuvant chemotherapy (temozolomide, TMZ) (Fekete et al., 2023; Sabouri et al., 2024). The blood-brain barrier (BBB), along with the high heterogeneity of GB, extensive diffuse infiltration, robust vascularization to sustain tumor growth, and tissue necrosis coupled with inflammatory microenvironment, often result in incomplete surgical resection and multidrug resistance (MDR), ultimately leading to tumor recurrence, at which point none of the available therapies can effectively extend the patient’s survival (Couto et al., 2019; Fanfone et al., 2020; Magalhães et al., 2021).

Inflammation, recognized as a pivotal hallmark of cancer, plays a crucial role in promoting tumorigenesis, with chronic inflammation frequently tangled with tumorigenic processes (Fouad and Aanei, 2017). Chronic inflammatory processes influence all stages of tumor development and treatment. In addition to tumor initiation, inflammation is decisive in tumor promotion, malignant conversion, and metastatic dissemination (Elinav et al., 2013). Neuroinflammation, an innate immune response to pathological stimuli within the CNS, significantly contributes to tumorigenesis of GB by promoting tumor growth and invasion. GB, referred to as a “cold tumor,” exhibits an immunosuppressive profile, generating pro-inflammatory cytokines (e.g., interleukin (IL)-6 and IL-8), thus fostering a state of chronic inflammation within the tumor microenvironment (TME) (Yeung et al., 2013; Todoric et al., 2016; Chen et al., 2022; Zhou et al., 2023). This complex and unique TME includes both tumor and non-tumor cells (such as endothelial and immune cells), along with cellular and non-cellular factors, and is responsible for regulating all molecular and cellular characteristics of the tumor and the surrounding tissue (Sharma et al., 2023). Considering this, several clinical trials were employed using immunotherapeutic strategies (e.g., immune checkpoint inhibitors, cancer vaccines, and oncolytic viral therapies) in combination with the standard of care for GB patients and observed a slight increase in the median OS (Liu et al., 2024). Thus, exploring innovative therapeutic strategies that target the interplay between neuroinflammation and the TME of GB emerges as an intriguing approach to inhibit the proliferation and invasion of this lethal brain tumor. Given the recognized anti-inflammatory and cytotoxic properties inherent in many plant-based bioactive compounds, the use of natural products represents a viable and appealing strategy (Apaya et al., 2016; Sun et al., 2023).

In this context, 7α-acetoxy-6β-hydroxyroyleanone (Roy), a naturally occurring abietane diterpene isolated from *Plectranthus hadiensis* Schweinf. (*P. hadiensis*), is a natural lead compound identified by our team for its significant cytotoxic and/or cytostatic activity across various cancer types (Matias et al., 2019; Śliwiński et al., 2020; Ntungwe et al., 2021; Magalhães et al., 2024). Moreover, in our previous work, we found that Roy exhibited a strong therapeutic effect across a panel of distinct GB cell lines, each representing a distinct molecular subtype of GB. Roy induced apoptosis through activation of the caspase cascade and inhibition of anti-apoptotic pathways. This natural lead compound also demonstrated a more effective cytotoxic activity than TMZ, the current first-line treatment, in these cells. For example, in U87 cells, Roy displayed a substantially lower IC_50_ value than TMZ (59.37 µM vs. 371.21 µM), as determined from side-by-side concentration–response curves, enabling direct comparison of their cytotoxic and antiproliferative effects (Magalhães et al., 2024). Consequently, the present study aims to investigate the antiproliferative and immunomodulatory potential of Roy, building on evidence that plant-based compounds, produced as defense mechanisms against environmental stressors and predators, often exhibit both immunomodulatory properties and inherent toxicity (Magalhães et al., 2021; Khairy et al., 2023; Moudgil and Venkatesha, 2023). Specifically, we will assess its ability to modulate the expression of key genes and secreted cytokines involved in neuroinflammation processes, tumor progression, and angiogenesis in a well-established GB cell line. Additionally, we will evaluate Roy’s effectiveness in reducing tumor size and metabolic activity in multicellular 3D models of GB.

## 2 Materials and methods

### 2.1 Plant material

*P. hadiensis* was provided by the Kirstenbosch National Botanical Gardens (Kirstenbosch, South Africa) and grown in Parque Botânico da Tapada da Ajuda (Lisbon, Portugal). The plant was air-dried at room temperature and stored in cardboard boxes protected from light and humidity to maintain its stability as previously described by our team (Domínguez-Martín et al., 2022; Magalhães et al., 2024).

### 2.2 Isolation of roy

**FIGURE 1:**
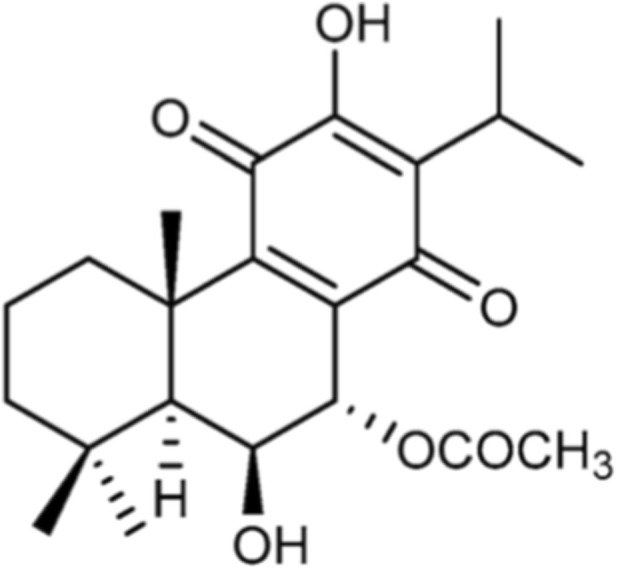
Chemical structure of 7α-acetoxy-6β-hydroxyroyleanone (Roy).

The abietane diterpene, Roy (Figure 1), was isolated from the acetonic extract of *P. hadiensis*, as previously described by our team (Domínguez-Martín et al., 2022; Magalhães et al., 2024). Roy was purified by dry-column flash-chromatography, using silica as a stationary phase and a gradient of eluents of increasing polarity (n-Hexane:Ethyl acetate) as a mobile phase. The structure of both Roy was elucidated by nuclear magnetic resonance (NMR) spectra assignments and previously published (Ntungwe et al., 2021).

### 2.3 Synthesis of Roy-Bodipy

Roy-Bodipy was synthesized following the protocol previously described by our team (Domínguez-Martín et al., 2022). Briefly, succinic anhydride was added to a mixture of BODIPY (fluorescent probe) and triethylamine in anhydrous dichloromethane. The reaction mixture was stirred at room temperature. The crude material of succinimidyl-modified BODIPY was employed in the subsequent stage without undergoing additional purification. A mixture of carboxylic acid (succinimidyl-modified BODIPY) and abietane in anhydrous dichloromethane was treated with 1-[3-(dimethylamino)propyl]-3-ethylcarbodiimide methiodide (EDC) and a catalytic amount of 4-(Dimethylamino)pyridine (DMAP). The solution was diluted and washed. The crude material was purified through silica column chromatography to yield Roy-BODIPY.

### 2.4 Cell culture

U87 (HTB-14; brain-likely GB) and HMC3 (CRL-3304; microglia (MG)) cell lines were acquired at American Type Culture Collection (ATCC) and provided by Prof. Carla Vitorino (Faculty of Pharmacy, University of Coimbra) and Prof. Célia Gomes (Coimbra Institute for Clinical and Biomedical Research (iCBR), Faculty of Medicine, University of Coimbra), respectively. Cells were cultivated in Dulbecco’s Modified Eagle’s Medium-high glucose (DMEM-HG) (Biowest, Nuaillé, France) supplemented with 10% (v/v) of heat-inactivated fetal bovine serum (FBS) (Biowest, Nuaillé, France) and 1% (v/v) of penicillin-streptomycin (Sigma, St. Louis, MO, United States). Human brain capillary endothelial cells (HBMEC) were purchased from Innoprot (Bizkaia, Spain) and kindly provided by Prof. Carla Vitorino (Faculty of Pharmacy, University of Coimbra). HBMEC were grown in EBM™-2 Basal Medium (CC-3156) and EGM™-2 SingleQuots™ Supplements (CC-4176) (Lonza Group AG, Basel, Switzerland) in T75 flasks coated with 0.3 mg/mL rat tail collagen type I (BD Biosciences). According to the supplier recommendations, HBMEC cells were used until a maximum of 15 passages, counting from the initial vial. All cells were maintained at 37 °C and 5% CO_2_ and subcultured by trypsinization when reaching 80%–90% confluence. All experiments were performed in cultures in log phase growth.

For all experiments, cells were treated with a single administration of 16 μM of Roy, a concentration previously shown in our work to exhibit cytotoxic effects in GB cells but not in non-tumoral cells (HMC3 cells and Primary cultures of astrocyte-enriched cells), indicating a tumor-specific cytotoxicity (Magalhães et al., 2024).

### 2.5 Assessment of metabolic activity and permeability studies through the blood-brain barrier

HBMEC cells (20 × 10^3^ cells/well) were seeded in a 96-well plate, 24 h before treatment. Following, cells were treated with 16 μM of Roy and further incubated for 4 h. Metabolic activity was assessed through a modified Alamar blue® assay, as previously described (Magalhães et al., 2024). Accordingly, 100 μL of a solution of EBM™-2 Basal Medium with 10% (v/v) of a stock solution of resazurin was added to the cells and incubated for 2 h, at 37 °C. The absorbance of the plate was read at 570 and 600 nm in a BioTek (BioTek Instruments, Inc., Winooski, VT, United States). The absorbance results were obtained by the Gen5 program. Metabolic activity was then calculated by Equation 1:

$$Metabolic\;activity\;\left(\%\right)=\frac{\left(A570-A600\right)\;of\;treated\;cells}{\left(A570-A600\right)\;of\;control\;cells\;}\times 100\%$$ (1)

To establish an *in vitro* model of the BBB, HBMEC cells (5 × 10^4^ cells/cm^2^) were seeded on the apical side of transwell inserts (in 6-multiwell collagen pre-coated Transwell®, 0.4 μm pore PTFE membrane insert (Corning, Glendale, AZ, United States) and grown for 7 days at 37 °C and 5% CO_2_. Cell medium was changed every 2 days. The formation of tight junctions (TJ) was monitored by measuring the transendothelial electrical resistance (TEER) of the cell monolayer by using an EVOM resistance meter (World Precision Instruments, Hertfordshire, UK). On day 7 of HBMEC culture in the transwell system, 16 µM of Roy-Bodipy was added to the apical (upper) compartment of the transwell. After 0.5, 1, 2, and 4 h, the medium in the basolateral (lower) compartment was collected and analyzed in a BioTek device (BioTek Instruments, Inc., Winooski, VT, United States) by measuring fluorescence at excitation and emission wavelengths 485 and 528 nm, respectively, for Roy-Bodipy determination. The initial concentration of Roy-Bodipy (16 µM) added to the upper compartment was considered as total of Roy-Bodipy, while the final concentration of Roy-Bodipy measured in the lower compartment was expressed as the percentage of Roy-Bodipy in the apical compartment at time 0. Permeability under steady-state conditions can be evaluated mathematically by the apparent permeability coefficient (Papp), according to Equation 2:

$$P_{app}=\frac{dQ}{dt}\frac{1}{AC_{0}}$$ (2)

where dQ/dt is the solute flux (μg/s) across the barrier, A is the surface area of the transwell (cm^2^), and C_0_ is the initial donor concentration (μg/mL). Whereas transport efficiency (TE) of Roy-Bodipy across the membrane was measured using Equation 3, as described by Pogačnik et al. (2016):

$$TE=\frac{Clow}{Cup}\times 100\%$$ (3)

where C_low_ is the concentration of Roy-Bodipy in the basolateral compartment and C_up_ is the concentration of Roy-Bodipy in the upper compartment, at the end of the experiment.

The permeability of sodium fluorescein (Na-F) (376.27 Da) was measured to confirm the integrity of the membrane as previously described (Pogačnik et al., 2016). 10 μg/mL of a Na-F solution, pH 7.4, was added to the upper compartment for 1 h. After 30 and 60 min, the medium in the lower chamber was collected and the concentration of Na-F was determined by fluorescence (excitation, 440 nm; emission, 525 nm) using a BioTek device (BioTek Instruments, Inc., Winooski, VT, United States).

### 2.6 3D spheroids: generation and treatment

Spheroids were grown in mono-, dual-, and triple-culture using U87 cells, U87 and HMC3 cells, and U87, HMC3, and HBMEC cells, respectively. For mono-culture, U87 cells were seeded at a density of 8 × 10^3^ cells per well, while for dual-culture and triple-culture, cells were seeded at a 1:1 ratio (U87:HMC3) and 2:1:0.2 ratio (U87:HMC3:HBMEC) per well, respectively, in ultra-low adherence round-bottomed 96-well plates (Corning, Glendale, AZ, United States) and further incubated for 72 h (Pires et al., 2012; Martins et al., 2023). At least 10 spheroids were generated per condition. After aggregation, spheroids were treated with a single administration of 16 µM of Roy, and media was replaced every 2 days for a total duration of 14-day treatment.

### 2.7 Analysis of 3D spheroids: metabolic activity, size, and circularity characterization

Spheroids were imaged, at days 0, 7, and 14, using the Motic Images Plus 3.0 (Barcelona, Spain), and spheroid size and circularity were determined using the ImageJ/Fiji software (NIH, United States) (Pires et al., 2012; Martins et al., 2023). For metabolic activity assessment, at day 14, spheroids were incubated with a solution of DMEM-HG with 10% (v/v) of a resazurin stock solution (Sigma. St. Louis, MO, United States) (0.1 mg/mL) for 4 h at 37 °C. After incubation, the absorbance of the plate was read at 570 and 600 nm in a BioTek (BioTek Instruments, Inc., Winooski, VT, United States). Metabolic activity was calculated using Equation 1.

### 2.8 Conditioned medium: assessment of metabolic activity and cell morphology

U87 and HMC3 cells (75 × 10^3^ cells/well) were seeded in 12-well plates, while co-culture of U87 and HMC3 cells was seeded at a final density of 15 × 10^4^ cells per well in 12-well plates. After 24 h, the medium was replaced by fresh medium, and cells were further incubated for 48 h. Secretome from the different cultures was collected and centrifuged at 1,500 rpm for 5 min, to remove potential cell debris. The conditioned medium was used in the metabolic activity assay.

U87 cells (1 × 10^4^ cells/well) were seeded in 96-well plates, 24 h before treatment. Cells were pre-incubated with non-diluted conditioned medium from U87 and/or HMC3 for 1 h. Subsequently, the conditioned medium was removed, and cells were treated with 16 µM of Roy and further incubated for 48 h. After treatment, the medium containing Roy was completely removed to minimize potential direct chemical reduction of resazurin by Roy. Metabolic activity of cells was assessed through a modified Alamar blue® assay as previously described. Briefly, a solution was prepared of DMEM-HG medium with 10% (v/v) of a resazurin salt dye stock solution at a 0.1 mg/mL concentration, which was further added to each well after 48 h treatment. After 4 h of incubation at 37 °C and 5% CO_2_, the absorbance of the plate was read at 570 and 600 nm in a BioTek (BioTek Instruments, Inc., Winooski, VT, United States). The absorbance results were obtained by the Gen5 program. Metabolic activity was determined using Equation 1.

Cell morphology was assessed using an inverted microscope Motic AE2000 (Barcelona, Spain) and images were obtained using the Motic Images Plus 3.0 (Barcelona, Spain).

### 2.9 Cytokines analysis

Cytokines were quantified using a customized detection panel purchased from Bio-techne (R&D Systems, Inc., Minneapolis, MN, United States), as previously described (Valerii et al., 2021). Briefly, U87 cells (40 × 10^3^ cells/well) were seeded in 24-well plates. After 24 h, cells were treated with 16 µM of Roy and, further, incubated for 48 h. The supernatant was collected and centrifuged at 4000 RCF for 10 min. After centrifugation, the supernatant was collected into new tubes and stored at −80 °C until use.

The inflammatory cytokines evaluated were IL-1β, IL-6, TNFα, IL-4, IL-8, and IL-10. The assay was performed in 96-well filter plates by multiplexed Luminex® (Luminex Corp., Austin, TX, United States) immunoassay according to the manufacturer’s instructions. Microsphere magnetic beads coated with monoclonal antibodies against the different target analytes were added to the wells and incubated for 30 min. After, the wells were washed and biotinylated secondary antibodies were added. After 30 min, beads were washed and then incubated for another 10 min with streptavidin-PE conjugated to the fluorescent protein, phycoerythrin (streptavidin/phycoerythrin). After washing, the beads (a minimum of 100 per analyte) were analyzed in the BioPlex 200 instrument (BioRad®, Hercules, CA, United States). Sample concentrations were estimated from the standard curve using a fifth-order polynomial equation and expressed as pg/mL (Supplementary Figure S1) after adjusting for the dilution factor (Bio-Plex Manager software 5.0). Samples below the detection limit of the assay were recorded as zero, while samples above the upper limit of quantification of the standard curves were assigned the highest value of the curve.

### 2.10 Quantitative real-time PCR

Expression levels of *Vascular Endothelial Growth Factor A (VEGFA), Signal Transducer and Activator Of Transcription 3 (STAT3), STAT5A, STAT5B, Janus kinase 2 (JAK2), IL6, IL8* and *Cyclin-dependent kinase 4 (CDK4)* mRNA were assessed by quantitative real-time PCR (qRT-PCR). U87 cells were seeded with a density of 1 × 10^6^ cells per well 24 h before treatment. After, cells were treated with 16 µM of Roy, and further incubated for 48 h. Total RNA was extracted using TripleXtractor solution (GRISP, Lisbon, Portugal) according to the manufacturer’s protocol. RNA was converted into cDNA through the Xpert cDNA Synthesis Supermix (GRISP, Lisbon, Portugal), following the manufacturer’s protocol and 100 ng of cDNA were amplified by qRT-PCR using the primer sequences described in Supplementary Table S1. Each optimized reaction was performed using Xpert Fast SYBR Green Mastermix 2X with ROX (GRISP, Lisbon, Portugal) and samples were subjected to the amplification protocol described by the manufacturer, using a melting temperature of 60 °C. Relative gene expression was determined by the 2^−ΔΔCT^ method and normalized to the *glyceraldehyde-3-phosphate dehydrogenase (GAPDH)* gene, the endogenous reference, and relative to the untreated control cells. Optimization conditions are described in Supplementary Table S2.

### 2.11 Colony formation assay

U87 cells (0.8 × 10^3^ cells/well) were seeded in 6-well plates. After 24 h, cells were treated with 16 µM of Roy and further incubated for 14 days with the media being changed every 2 days. Non-treated cells were considered the control group. After, cells were washed with PBS, fixed with 4% PFA in PBS for 15 min, and then stained with 0.1% crystal violet solution for 30 min. The surviving fraction (SF) was calculated as mean colonies/number of cells seeded. A minimum of 50 cells was required to define a colony.

### 2.12 Statistical analysis

Data were analyzed using GraphPad Prism v.10.1.0. All experiments are representative of at least three independent experiments and acquired results were expressed as mean ± standard deviation (SD). Statistical analysis was performed by t-student test, one-way, and two-way ANOVA, using the unpaired comparison and the multiple comparisons tests Tukey and Dunnett, respectively. A value of *p* < 0.05 was considered significant.

## 3 Results

### 3.1 Assessment of Roy transport across the blood-brain barrier

**FIGURE 2:**
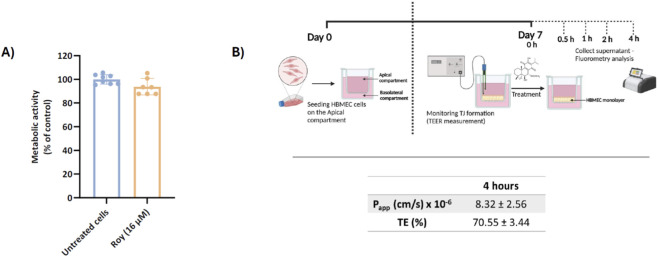
Assessment of Roy’s capability to cross a brain endothelial cell monolayer *in vitro*. **(A)** Effect of 16 µM Roy in the metabolic activity of HBMEC cells after a 4 h-treatment. Metabolic activity was evaluated using Alamar Blue® assay and results were expressed as percentage of control (untreated cells). **(B)** Scheme of the *in vitro* BBB model with HBMEC cells in a Transwell® system and assessment of the apparent permeability coefficient (Papp) value and transport efficiency (TE) of Roy-Bodipy (16 µM) using this BBB model. Data are presented as mean ± SD and it is representative of at least three independent experiments. Scheme was generated using objects from Biorender.

The BBB presents a significant challenge for treating GB, as it protects the brain from both exogenous and endogenous molecules, thereby hindering the entry and accumulation of most therapeutic agents in brain tissue, which results in lower treatment efficacy. In this study, we evaluated the transport efficiency of Roy across the BBB using the HBMEC model, after 4 h, and assessed its cytotoxicity, for the same incubation time, to ensure it does not damage the BBB cells (Figure 2). We used a concentration of 16 µM of Roy for the treatment, as, in our previous work, this concentration had previously demonstrated an antitumor effect on GB cells while being safe for non-tumor cells (MG and astrocytes) (Magalhães et al., 2024). Our findings indicated that this concentration of Roy did not cause damage to HBMEC cells, being considered safe (Figure 2A). Subsequently, we investigated Roy’s ability to cross the BBB *in vitro*. For this purpose, Roy was conjugated to the fluorescent probe Bodipy (Roy-Bodipy), enabling its quantification by fluorescence. Notably, conjugation with Bodipy did not alter Roy’s bioactivity (Data not shown). We used TEER and Na-F as markers to confirm BBB integrity. The permeability value of Na-F below 1.2 × 10^−3^ cm/min indicated no adverse effect on the cell monolayer integrity, which was confirmed by TEER values averaging 140–150 Ω.cm^2^. The apparent permeability of Roy (Papp = 8.32 × 10^−6^ cm/s) and the effective concentration of this natural compound that permeated the membrane (expressed as TE = 70.55%) (Figure 2B) demonstrated Roy’s ability to cross the BBB.

### 3.2 Effect of Roy in 3D multicellular spheroids of glioblastoma microenvironment

To assess the effectiveness of Roy treatment in cell models that better mimic the TME, 3D multicellular spheroid models were employed. These models are widely recognized *in vitro* tools for investigating innovative therapeutic strategies against cancer due to their ability to simulate the TME *in vitro* (Pires et al., 2012; Zanoni et al., 2016). In this study, we generated a heterogeneous 3D spheroid model of GB microenvironment, incorporating its three main cellular components: tumor cells, immune cells, and endothelial cells. In primary GBs, immune cells can represent 30%–50% of the tumor mass and play a crucial role in promoting tumor proliferation, angiogenesis, and immune suppression (Larionova et al., 2021; Li et al., 2022; Chen et al., 2022). Endothelial cells, which constitute approximately 5%–30% of the tumor mass, are key mediators of tumor angiogenesis, being involved in the modulation of the pro-tumor immune response and therapeutic resistance (Martins et al., 2023; Leone et al., 2024). This 3D model therefore allowed us to assess the efficacy of Roy treatment within a more realistic tumor structure. To represent tumor cells, the well-characterized human GB cell line U87 was chosen, as recommended by ATCC and supported by our team’s prior experience using this model to develop GB spheroid systems (Pires et al., 2012). Additionally, HMC3 cells, a human MG cell line, and HBMEC, human brain microvascular endothelial cells, were selected to represent the immune and endothelial components, respectively, in this model.

**FIGURE 3:**
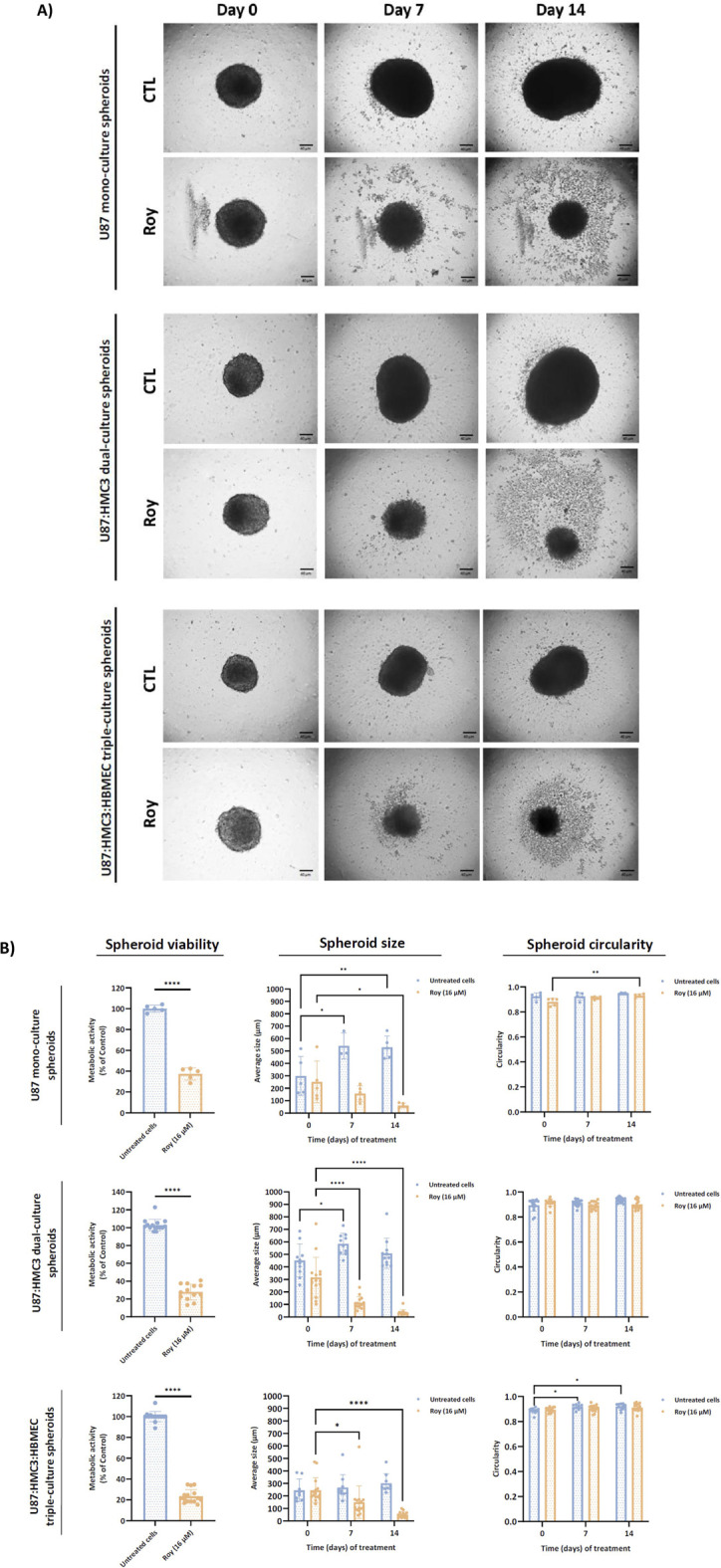
Effect of treatment with Roy on U87 mono-culture spheroids, U87:HMC3 dual-culture spheroids, and U87:HMC3:HBMEC triple-culture spheroids. After 72 h, 3D spheroids were treated with 16 µM of Roy and further incubated for 14 days, with media being refreshed every 2–3 days. **(A)** Representative images of treated spheroids at days 0, 7, and 14 are displayed. Images were obtained by an inverted microscope with a total ampliation of 40X. **(B)** The mean spheroid size and circularity progression were measured on days 0, 7, and 14. Metabolic activity was evaluated at day 14 and expressed as percentage of control. Untreated spheroids were considered controls. Asterisks (*p < 0.05, **p < 0.01, and ****p < 0.0001) represent the values that significantly differ from the control condition. Statistical analyses were performed to compare each treated condition with the corresponding control.

Monocellular and multicellular spheroids were formed and treated with 16 µM of Roy for 14 days. We monitored spheroid size and circularity during this period (Figure 3B). Notably, treatment with Roy significantly reduced spheroid size after 7 and 14 days of treatment in multicellular spheroid cultures, whereas this significative effect was only observed after 14 days in mono-culture spheroids (Figure 3A). However, we found no significant differences in spheroid circularity after treatment (Figure 3B). Moreover, at day 14, treated spheroids exhibited a substantial decrease in metabolic activity and a marked reduction in size, accompanied by apparent structural changes, when compared to untreated spheroids, despite overall spheroid circularity being preserved (Figure 3). This underscores Roy’s strong cytotoxic and potential cytostatic effects, as well as, its potential to mitigate the inflammatory state of the GB microenvironment, demonstrating greater efficacy in complex tumor structures, reinforcing its promise as a natural lead compound.

### 3.3 Effect of Roy in the metabolic activity of glioblastoma cells pre-incubated with conditioned medium

**FIGURE 4:**
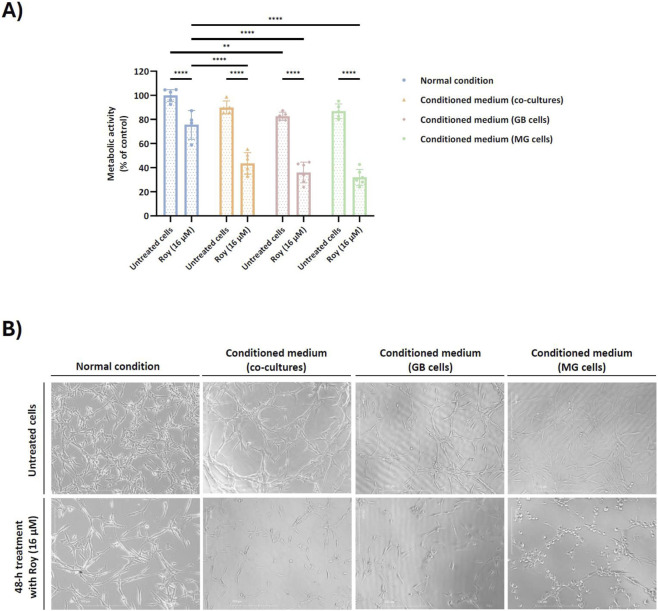
Effect of conditioned medium on the cytotoxic/antiproliferative activity of Roy in glioblastoma cells. U87 cells were pre-incubated for 1 h either with secretome from untreated cells, namely co-cultures (U87 and HMC3 cells), culture of U87 cells, or culture of HMC3 cells. After, cells were treated with 16 µM of Roy for 48 h. **(A)** Metabolic activity was assessed through Alamar blue® assay and was expressed as percentage of control (untreated cells in the normal condition). Asterisks (**p < 0.01, and ****p < 0.0001) represent the values that significantly differ from the other conditions. Statistical analyses were performed to compare each treated condition with the untreated cells in the normal condition. Data are presented as mean ± SD and it is representative of at least three independent experiments. **(B)** Representative images of treated cells after 48 h are displayed. Images were obtained by an inverted microscope with a total ampliation of 40X.

As previously noted, Roy demonstrated the ability to inhibit the growth of tumor spheroids over time with a nearly complete spheroid disintegration by day 14 (Figure 3). Considering these findings, we intended to determine whether this lead compound could maintain its cytotoxic/antiproliferative activity in the presence of conditioned medium. To investigate this, we exposed GB cells to conditioned medium derived from cultures of GB cells, MG cells, or co-culture of GB and MG cells, and subsequently treated them with 16 µM of Roy (the same concentration used in the previous assays) (Figure 4). The results showed that Roy’s effectiveness increased, leading to a significant reduction in the metabolic activity of GB cells when compared to its activity in the presence of fresh medium (Figure 4A). Moreover, in the presence of conditioned medium from MG cells, Roy treatment caused a 40% reduction in metabolic activity compared to the control group (non-treated cells) (Figure 4A). In contrast, under normal conditions (using fresh media), Roy induced only a 20%–30% decrease in metabolic activity relative to the control (Figure 4A). This data was supported by changes in the morphology of treated cells that match with cells undergoing apoptosis (Figure 4B). Interestingly, Roy’s antitumor effect on spheroids (Figure 3) appears to be linked to its improved efficacy in the presence of a complex TME.

### 3.4 Roy impact on cytokine levels secreted by glioblastoma cells

Various cytokines within the TME of GB play a crucial role in shaping immune and inflammatory responses, thus influencing tumor development, angiogenesis, and progression. Targeting signaling molecules associated with neuroinflammation processes may be a promising strategy to combat this invasive brain tumor. Building on our previous findings, where Roy demonstrated enhanced efficacy in models that better mimic the TME (Figures 3, 4), and considering the critical role of cytokines in shaping the GB microenvironment, we evaluated the impact of Roy treatment on the expression of cytokines secreted by GB cells that are involved in the tumorigenesis process.

**FIGURE 5:**
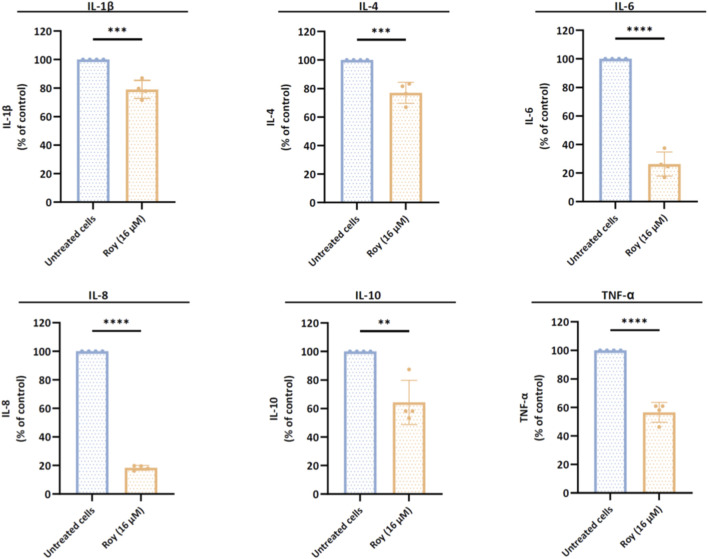
Assessment of cytokines levels released by glioblastoma cells after treatment with Roy. U87 cells were treated with 16 µM of Roy and incubated for 48 h. After incubation, the secretome of treated and untreated cells was collected and analyzed by multiplexed Luminex® immunoassay. The expression of IL-1β, IL-4, IL-6, IL-8, IL-10, and TNF-α was normalized to CTL (untreated cells) and expressed as percentage of the control. Asterisks (**p < 0.01, ***p < 0.001, and ****p < 0.0001) represent the values that significantly differ from the control (untreated cells). Statistical analyses were performed to compare each treated condition with the corresponding control. Data are presented as mean ± SD and it is representative of at least four independent experiments.

Our findings suggested that Roy exhibited an immunomodulatory effect on GB cells, significantly reducing the levels of several cytokines involved in the progression of neuroinflammation, angiogenesis, and tumor growth, namely IL-1β, IL-6, IL-8, and TNF-α (Figure 5). Additionally, Roy treatment also led to a decrease in IL-4 and IL-10 levels, cytokines with a dual role, typically anti-inflammatory but seemingly pro-inflammatory in the context of GB (Figure 5). Clearly, Roy also displayed an immunomodulatory impact, reinforcing its therapeutic potential as observed in our previous experiments.

### 3.5 Effect of Roy on the expression of mRNA intracellular levels of genes associated with IL-6 pathway

**FIGURE 6:**
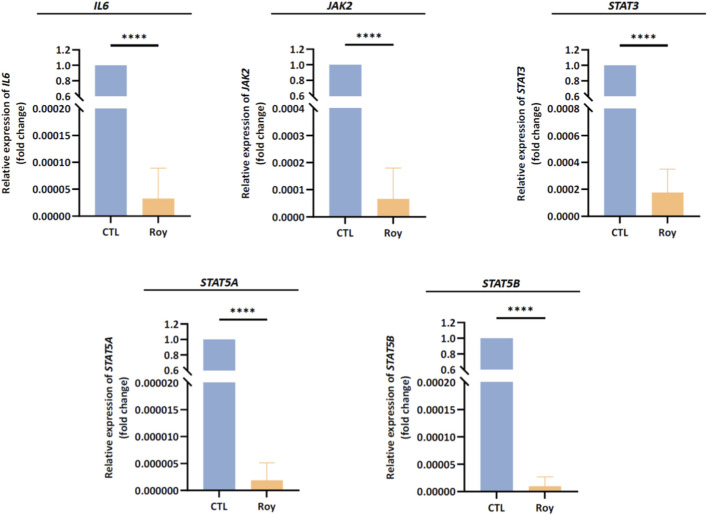
Assessment of *IL6, JAK2, STAT3, STAT5A,* and *STAT5B* mRNA levels in U87 cells after treatment with Roy. Cells were treated with 16 µM of Roy and further incubated for 48 h. After incubation, the relative expression of *IL6, JAK2, STAT3, STAT5A,* and *STAT5B* mRNA levels was assessed by qRT-PCR, and the results were normalized to *GAPDH* expression. Asterisks (****p < 0.0001) represent the values that significantly differ from the control (untreated cells). Statistical analyses were performed to compare each treated condition with the corresponding control. Data are presented as mean ± SD and it is representative of three independent experiments.

Based on the previous findings, we observed that Roy exhibited a strong antitumor activity, inhibiting tumor growth and possibly modulating immune-related targets. With this in mind, we assessed the mRNA levels of genes linked to the IL-6/JAK2/STAT3 signaling pathway, known to be overexpressed in GB and associated with uncontrolled proliferation, invasion, and survival. Following a 48-h treatment period with Roy, we analyzed the transcriptional profiles of *IL6*, *JAK2*, *STAT3*, *STAT5A*, and *STAT5B* in GB cells (Figure 6). Notably, we observed a significant downregulation of *IL6*, *JAK2*, *STAT3*, *STAT5A*, and *STAT5B* mRNA levels, all of which are found to be upregulated in GB cells, compared to the control group (untreated cells) (Figure 6). These findings align with the observed inhibition of GB immune-endothelial spheroids size (Figure 3) and the reduction in IL-6 levels secreted by GB cells (Figure 5), further supporting that Roy potentially suppresses IL6/JAK2/STAT3 pathway activity, consequently inhibiting GB proliferation and survival.

### 3.6 Roy effect on clonal growth and mRNA levels of genes associated with proliferation mechanisms

**FIGURE 7:**
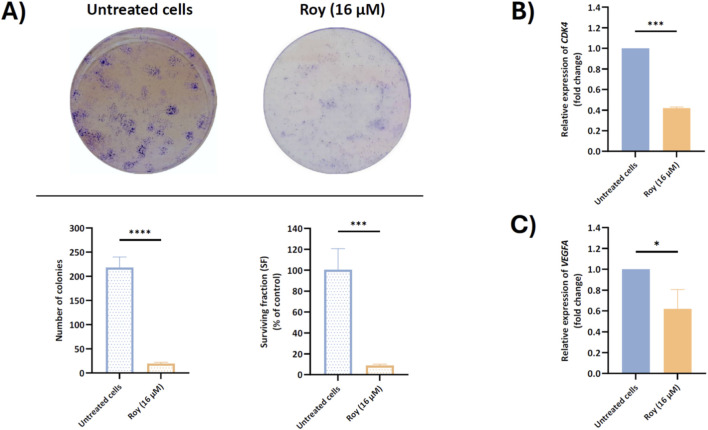
Assessment of colony formation **(A)** and *CDK4* **(B)** and *VEGFA* **(C)** mRNA levels in U87 cells, after treatment with Roy. Cells were treated with 16 µM of Roy. **(A)** After a 14-day incubation, colony formation was assessed by microscopy and the number of colonies and surviving fraction was quantified using FIJI/imagej. **(B,C)** After 48 h, the relative expression of *CDK4* and *VEGFA* mRNA levels was assessed by qRT-PCR, and the results normalized to *GAPDH* expression. Asterisks (*p < 0.05, ***p < 0.001, and ****p < 0.0001) represent the values that significantly differ from the control (untreated cells). Statistical analyses were performed to compare each treated condition with the corresponding control. Data are presented as mean ± SD and it is representative of at least three independent experiments.

Considering the reduction in spheroid size following Roy treatment (Figure 3) and its effects on immune-related targets implicated in GB proliferation and invasion (Figures 5, 6), we next explored the antiproliferative activity of this lead compound on GB clonal growth (Figure 7A). By the results obtained, we observed that treatment with Roy led to almost complete impairment of colony formation as sustained by the reduced surviving fraction (SF) factor (Figure 7A). Based on this effect, significant inhibition of *CDK4* mRNA levels, a gene encoding Cdk4 an important regulator of cell cycle progression, following Roy treatment was also observed (Figure 7B), supporting the antiproliferative and antitumor effect of this lead compound. Moreover, besides uncontrolled proliferation, GB is also characterized by extensive vascularization, leading to robust angiogenesis. Building upon our previous findings, it was also investigated how treatment with Roy affects the expression of *VEGFA* mRNA levels. As anticipated, Roy induced downregulation of *VEGFA* mRNA levels in GB cells (Figure 7C). These results align with the antitumor potential of Roy previously observed, suggesting an antiproliferative effect and ability to impair tumor growth and promote cell death.

## 4 Discussion

Medicinal plants offer a rich source of unique bioactive compounds, exhibiting remarkable therapeutic potential, including antitumor, cytotoxic, antiproliferative, and anti-inflammatory properties. They have been used since ancient times to treat various illnesses, underscoring their invaluable contribution to the field of medicine and drug discovery (Rodrigues et al., 2016; Garcia-Oliveira et al., 2021; Khan et al., 2022). Notably, the immunomodulatory capabilities of natural compounds have drawn the attention of researchers within ethnomedicine (Jantan et al., 2015; Orhan et al., 2016; Shukla et al., 2022; Zebeaman et al., 2023). This is particularly significant due to the intimate connection between TME and inflammation, a hallmark of cancer (Hanahan and Weinberg, 2011; Fouad and Aanei, 2017). TME acts as a host that supports tumor growth and invasion, facilitates neoplastic transformation, and provides a proper environment for dormant metastases to succeed. This complex scene arises from interactions between malignant and non-transformed cells. The intricate heterogeneity within the TME is a major factor contributing to the disappointing outcomes in the GB treatment. GB has the capacity to manipulate the immune response by transforming it into a chronic inflammatory state. This promotes tumor relapse and triggers processes such as epithelial-mesenchymal transition (EMT) and MDR. GB cells release immune-suppressive cytokines that foster abnormal activation of inflammatory pathways, subsequently stimulating proliferation, migration, and angiogenesis (Al-kharboosh et al., 2020; Chen et al., 2022; Erices et al., 2023; Giles et al., 2023). In this context, innovative therapeutic approaches based on natural lead compounds present a promising option to address the challenge of GB. A previous study from our group unveiled the potential antitumor mechanism of Roy, an abietane diterpene isolated from *P. hadiensis*, in various GB cell lines representing the distinct molecular subtypes of this tumor (proneural, classical, and mesenchymal) (Magalhães et al., 2024). This natural lead compound induced cell cycle arrest and apoptosis via caspase activation in GB cells, along with mitochondrial fragmentation, upregulation of tumor suppressor genes, and inhibition of anti-apoptotic proteins, highlighting its therapeutic potential (Magalhães et al., 2024). Therefore, in this study, we evaluated the antiproliferative/antitumor and potential immunomodulatory effects of this compound (Roy) at 16 μM, the concentration previously identified as tumor-specific, in 3D and 2D cell models of GB, respectively.

As expected, Roy treatment significantly decreased the metabolic activity of 3D GB cell models and markedly reduced tumor size, leading to nearly complete disaggregation of the spheroids by day 14. This cytotoxic effect was particularly pronounced in a 3D GB immune-endothelial cell model, showing a substantial reduction in spheroid size by day 7. Interestingly, Roy’s efficacy appeared higher in more accurate models of the TME, as these 3D multicellular models better mimic tumor heterogeneity, cellular behavior *in vivo*, and cell-cell and cell-matrix interactions (Lu and Stenzel, 2018; Martins et al., 2023). Additionally, in GB cells pre-incubated with conditioned medium (secretome from either GB and/or MG cells), the cytotoxic and/or antiproliferative activity of Roy was enhanced, being this effect more pronounced in the condition pre-treated with secretome from MG cells. Morphological changes observed in this condition, including loss of intercellular connection, cell shrinkage, membrane blebbing, and fragmentation into membrane-bound vesicles, are hallmarks of apoptosis (Elmore, 2007). These effects may be related with soluble components (e.g., cytokines, chemokines, nitric oxide (NO)) secreted by MG cells, which could activate pro-apoptotic signaling pathways, thereby potentiating the antitumor effect of Roy and leading to a more substantial decrease in GB cells viability (Hwang et al., 2009; Matias et al., 2018). Although resazurin-based assays may be influenced by redox-active compounds, the assay in this study was conducted in the absence of Roy. Moreover, the concordance between the observed decrease in cells’ metabolic activity and the near-complete loss of colony-forming ability in the clonogenic assay following treatment with Roy supports that this compound’s cytotoxic/antiproliferative effects reflect a genuine impairment of long-term proliferative capacity, rather than assay-specific interference. While Roy contains a quinone moiety, a structural motif sometimes associated with pan-assay interference (PAINS), this feature is a well-established pharmacophore in abietane diterpenoids, consistently linked to apoptosis induction, mitochondrial dysfunction, and regulated DNA damage in cancer models. Importantly, the biological effects of Roy were consistently observed across multiple orthogonal assays and independent endpoints, supporting a regulated cellular response rather than nonspecific redox-driven artifacts (Sitarek et al., 2020; Śliwiński et al., 2020).

This significant antitumor activity aligns with an immunomodulatory effect exerted by Roy in GB cells. Treatment with Roy significantly reduced the levels of pro-inflammatory cytokines secreted by GB cells, which function in both autocrine and paracrine ways, contributing to a chronic inflammatory state that promotes tumor growth (Kartikasari et al., 2021). Cytokines such as IL-1β, IL-6, IL-8, and TNF-α are upregulated in GB cells and are associated with a poorer prognosis in patients. These cytokines collaboratively promote inflammatory gene expression, foster a proinflammatory phenotype in GB stem cells (GSCs), and activate inflammatory signaling cascades, leading to immune suppression, tumor growth, and invasion (Yeung et al., 2013; Alghamri et al., 2021; Garlanda and Mantovani, 2021; Hirano, 2021; Kartikasari et al., 2021).

Moreover, the inhibition of IL-1β and TNF-α secreted levels following Roy treatment likely contributed to the suppression of GB cell proliferation, migration, and angiogenesis, as evidenced by the significant decrease in IL-6 and IL-8 secreted levels and the downregulation of *VEGFA*, *IL6*, *JAK2*, *STAT3*, and *STAT5* intracellular mRNA levels (Yeung et al., 2013; West J. et al., 2018; Ou et al., 2021; Moyama et al., 2022). IL-1β and TNF-α stimulate the production of IL-6 and IL-8, which further drive GB proliferation and neovascularization. IL-6 and IL-8 are highly expressed in GB cells and are crucial in promoting proliferation, invasion, and angiogenesis (Yeung et al., 2013). Additionally, IL-6 overexpression upregulates the *STAT3* gene through activation of the *JAK2* signaling pathway. STAT3 is known for its aberrant signaling and predominant oncogenic role in GB, promoting invasion, proliferation, angiogenesis, and inhibition of apoptosis (West J. et al., 2018; Hirano, 2021; Ou et al., 2021). Similarly, IL-8 has a potent pro-angiogenic effect during tumorigenesis, inducing the upregulation of pro-invasion and pro-survival pathways in this malignant tumor (Dwyer et al., 2012; Lv et al., 2023). Moreover, IL-8 overexpression in GB is also associated with high levels of *VEGFA* mRNA, a gene encoding VEGF, a major player in angiogenesis and vascularization. Although the mechanisms and functional role of STAT5 activation in GB progression remain poorly understood, recent evidence indicates that STAT5 signaling contributes to GB tumorigenesis, promoting tumor invasion and survival (Ou et al., 2021; Blomquist et al., 2022; Blomquist et al., 2024; Moyama et al., 2022; Huang et al., 2024). In light of these findings, it becomes evident that Roy offers an attractive therapeutic effect by modulating the expression of pro-inflammatory cytokines secreted by GB cells, supporting our previous data on the antitumor potential of this compound (Magalhães et al., 2024). This may indicate a reversal of the tumorigenic process, attenuation of GB invasiveness and proliferation, and suppression of primary regulators of neuroinflammation in GB (Al-kharboosh et al., 2020; Alghamri et al., 2021; Basheer et al., 2021).

Interestingly, treatment with Roy also led to a reduction in IL-4 and IL-10 levels. IL-4, a known anti-inflammatory cytokine, usually plays a role in allergy-related physiological events. However, its role in brain injuries is controversial, and in the context of GB, it appears to have a pro-inflammatory effect, being secreted by GB cells at chronic levels, potentially explaining the infrequent occurrence of allergies in GB patients. However, this mechanism is not completely understood (Gadani et al., 2012). Similarly, IL-10, an anti-inflammatory cytokine, takes on a pro-inflammatory role within the GB microenvironment. Elevated IL-10 levels secreted by GB cells are linked to high malignancy and poor prognosis. In GB, IL-10 released by tumor cells may activate tumor-infiltrating immune cells, which in turn produce more IL-10 within the tumor tissue. Furthermore, high IL-10 levels can activate the JAK-STAT3 pathway, inhibiting apoptosis and promoting tumor proliferation, migration, and angiogenesis (Widodo et al., 2021).

Furthermore, it is important to note that the transcriptional changes observed for *STAT3* and *STAT5* genes suggest modulation of the JAK/STAT signaling axis. However, direct biochemical inhibition, protein-level regulation, and structural ligand–target interactions were not assessed in the present study and will require further investigation.

Additionally, it is noteworthy that this natural lead compound was able to cross an *in vitro* brain endothelial cell monolayer, an established model of the BBB, which represents a major obstacle in the effective treatment of GB. This was anticipated since Roy’s chemical properties adhere to Lipinski’s rule of five: it has a molecular weight under 400 Da, a log P value under 5, and fewer than 5 hydrogen bond donors (HBD) and 10 hydrogen bond acceptors (HBA) (Lipinski et al., 2001; Sakiyama et al., 2021).

However, recognizing the limitations of an entirely *in vitro* study and the use of a single GB cell line is crucial, as it introduces potential biases such as sampling bias, cross-contamination, and concerns regarding the authenticity of cell lines. Furthermore, GB is a highly heterogeneous disease, and no single model fully captures its biological complexity. To mitigate these challenges, cell lines were sourced from reputable suppliers, and multicellular 3D spheroid models were generated to better simulate the TME. This approach provides a more accurate *in vitro* model for testing our compound’s efficacy while addressing the difficulty of obtaining primary cultures, which rely on brain surgery samples. We also took steps to minimize other biases, including researcher bias, where prior knowledge and expectations might inadvertently influence data interpretation. To counteract this, we used standardized culture protocols, meticulously tracked cell line history and experimental conditions, conducted experiments across diverse cell models (2D and 3D with more than one type of cell line), and validated findings through replication. Additionally, we emphasized transparency by sharing our data and methodologies, ensuring reproducibility, and subjecting our study to a thorough peer review. Future work should assess whether the observed effects of Roy are conserved across additional GB models.

Overall, the findings of this study, combined with our existing knowledge of Roy’s antitumor mechanism (Sitarek et al., 2020; Magalhães et al., 2024), suggest that this natural lead compound effectively targets multiple key players in signaling pathways involved in GB proliferation and angiogenesis, aggressiveness, and invasiveness (e.g., VEGF, CDK4/6 axis and IL-6 pathway). Furthermore, based on our current knowledge, treatment with Roy appears to be a promising approach for future strategies targeting tumor-associated microglia/macrophages (TAMs), key players in shaping the microenvironmental landscape of GB. Potentially, by modulating TAMs phenotype, Roy may be able to shape the tumor immune microenvironment in GB, inhibiting tumor progression and survival. Its significant antitumor activity highlights the need for further investigation as a potential therapeutic strategy for GB, since despite modest progress in GB patient survival, treatment outcomes have remained stagnant, underscoring the need for new and effective anticancer therapies. Future research should assess whether the observed transcriptional changes translate into functional alterations at the protein level, and further evaluate Roy’s effects *in vivo* and in pre-clinical models of GB and investigate its pharmacokinetics, biodistribution, and optimal administration to ensure its therapeutic potential. Herein, our work aims to lay the foundation for advancing GB treatment, ultimately improving patient outcomes and quality of life.

## 5 Conclusion

In summary, this study delves into Roy’s immunomodulatory and tumor-specific cytotoxic properties, particularly in its capacity to target key signaling molecules involved in the regulation of neuroinflammation in GB cells. Furthermore, it demonstrates its potential as a noteworthy candidate for a natural lead compound, meriting further exploration in the investigation of GB therapy.

## Data Availability

The raw data supporting the conclusions of this article will be made available by the authors, without undue reservation.
