# Supplementary material for: 7α-acetoxy-6β-hydroxyroyleanone (Roy) modulates IL-6/STAT3/JAK2 mRNA expression and suppresses tumor growth in glioblastoma cell models
Source: Front Pharmacol. 2026 Feb 13;17:1728792. doi: 10.3389/fphar.2026.1728792 (PMC12946039; doi:10.3389/fphar.2026.1728792)
Supplement: Supplementary file 1 [file DataSheet1.pdf]

Supplementary Material

1 Supplementary Figures and Tables

1.1 Supplementary Figure 1

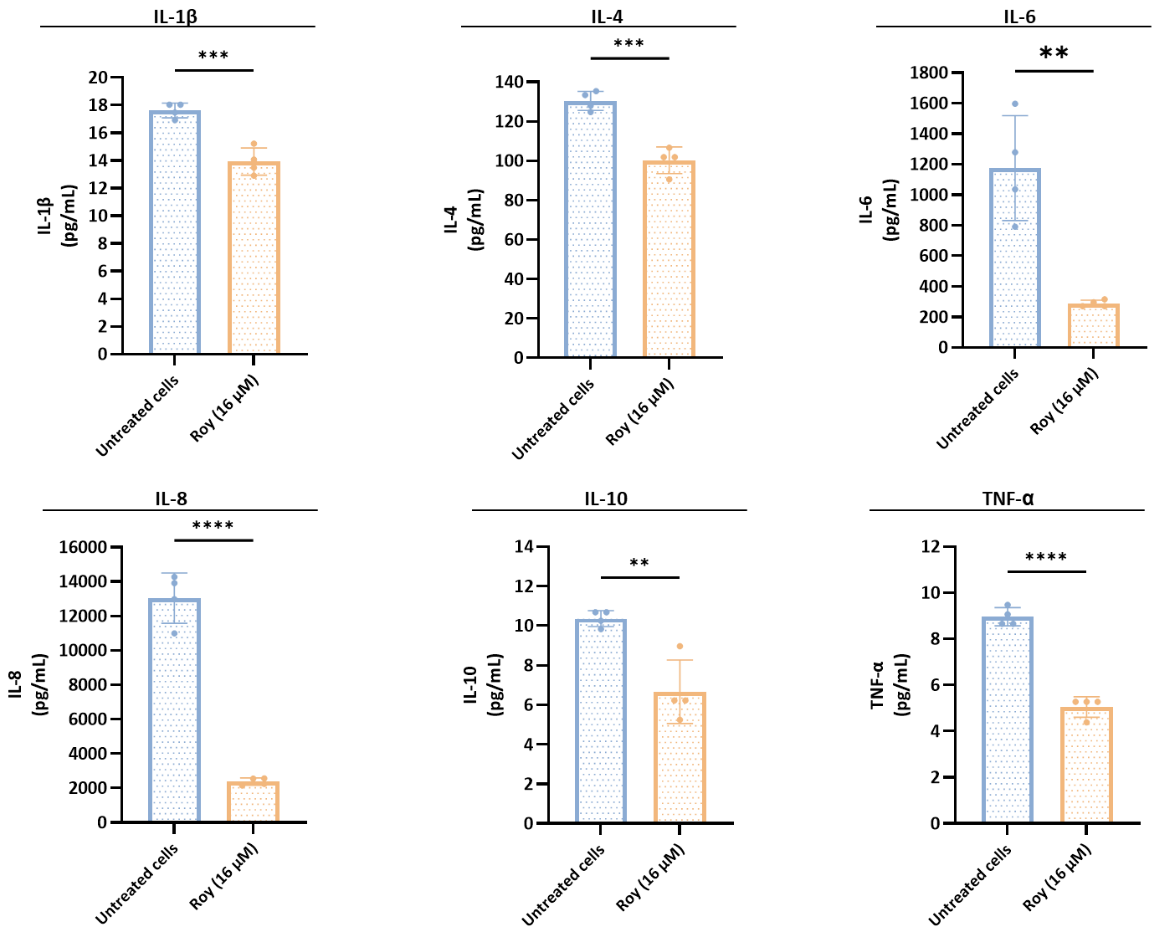

Supplementary Figure 1. Assessment of cytokines levels released by glioblastoma cells after treatment with Roy. U87 cells were treated with 16 μM of Roy and incubated for 48 h. After incubation, the secretome of treated and untreated cells was collected and analyzed by multiplexed Luminex® immunoassay. The expression of IL-1β, IL-4, IL-6, IL-8, IL-10, and TNF-α was estimated from the standard curve using a fifth-order polynomial equation and expressed as pg/mL. Asterisks (**p<0.01, ***p<0.001, and ****p<0.0001) represent the values that significantly differ from the control (untreated cells). Data are presented as mean ± SD and it is representative of at least four independent experiments.

1.2 Supplementary Table 1

Supplementary Table 1. Sequence of the primers and respective amplicon size, for each optimized reaction, in quantitative real-time PCR (qRT-PCR). Design and specificity of primers were performed using the Primer Blast tool (https://www.ncbi.nlm.nih.gov/tools/primer-blast/).

Gene	Amplicon length (bp)	Forward (5'-3')	Reverse (5'-3')
VEGFA	81	TGCAGATTATGCGGATCAAACC	TGCATTCACATTTGTTGTGCTGTAG
STAT3	176	ATCACGCCTTCTACAGACTGC	CATCCTGGAGATTCTCTACCACT
STAT5A	80	CGACGGGACCTTCTTGTTG	GTTCCGGGGAGTCAAACTTCC
STAT5B	130	GAACACCCGCAATGATTACAGT	ACGGTCTGACCTCTTAATTCGT
JAK2	130	TCTGGGGAGTATGTTGCAGAA	AGACATGGTTGGGTGGATACC
IL6	149	ACTCACCTCTTCAGAACGAATTG	CCATCTTTGGAAGGTTCAGGTTG
CDK4	128	AGCCGAAACGATCAAGGAT	GCTTGACTGTTCCACCACTTG
GAPDH	131	GTCTCCTCTGACTTCAACAGCG	ACCACCCTGTTGCTGTAGCCAA

1.3 Supplementary Table 2

Supplementary Table 2. Optimized conditions for each qRT-PCR reaction. **NOTE:** Primer concentration optimization was performed using calibration curves generated from cell lines exhibiting upregulation of the target genes, based on data from the Human Protein Atlas (https://www.proteinatlas.org/). Reaction efficiency for each assay was determined from the standard curves calculated by the equipment.

Gene	Primer concentration (nM)	Melting Temperature (ºC)	Efficiency (%)	Cell line	Enzyme / qPCR equipment
VEGFA	200	60	84.11	U87	Xpert Fast SYBR MDM2 Green Mastermix 2X with ROX / QuantStudio® 3 RealTime PCR Systems
STAT3	300	60	85.00	HEL	
STAT5A	200	60	94.98	K562	
STAT5B	200	60	85.77	MOLT-4	
JAK2	200	60	102.88	HEL	
IL6	200	60	99.88	U87	
CDK4	100	60	108.00	THP1	
GAPDH	150	60	92.15	NB4	
